# Effect of pneumoperitoneum pressure and the depth of neuromuscular block on renal function in patients with diabetes undergoing laparoscopic pelvic surgery: study protocol for a double-blinded 2 × 2 factorial randomized controlled trial

**DOI:** 10.1186/s13063-020-04477-x

**Published:** 2020-06-29

**Authors:** Xiaohan Xu, Yahong Gong, Yuelun Zhang, Jiaxin Lang, Yuguang Huang

**Affiliations:** 1 Department of Anesthesiology, Chinese Academy of Medical Sciences & Peking Union Medical College Hospital, Beijing, 100730 China; 2 Department of Medical Research Center, Chinese Academy of Medical Sciences & Peking Union Medical College Hospital, Beijing, 100730 China

**Keywords:** Diabetes, Pneumoperitoneum pressure, Neuromuscular block, Renal injury, Renal protection, Laparoscopic surgery

## Abstract

**Background:**

Patients with diabetes mellitus are at a high risk of developing postoperative acute kidney injury. For patients receiving laparoscopic surgery, standard-pressure pneumoperitoneum (SPP) currently applied in clinical practice also undermines renal perfusion. Several studies have shown that low-pressure pneumoperitoneum (LPP) might reduce pressure-related ischemic renal injury. However, LPP may compromise the view of the surgical field. Previous studies have indicated that deep neuromuscular blockade (NMB) can ameliorate this issue. However, the conclusion is still uncertain. The hypothesis of this study is that the joint use of LPP and deep NMB can reduce perioperative renal injury in diabetic patients undergoing laparoscopic pelvic surgery without impeding the view of the surgical field.

**Methods:**

This is a double-blinded, randomized controlled trial using a 2 × 2 factorial trial design. A total of 648 diabetes patients scheduled for major laparoscopic pelvic surgeries at Peking Union Medical College Hospital will be randomized into the following four groups: SPP (12–15 mmHg) + deep-NMB (post-tetanic count of 1–2) group, LPP (7–10 mmHg) + deep-NMB group, SPP + moderate-NMB (train-of-four of 1–2) group, and LPP + moderate-NMB group. The primary outcome is serum cystatin C level measured before insufflation, after deflation, 24 h postoperatively, and 72 h postoperatively. The secondary outcomes are serum creatinine level, intraoperative urine output, erythrocytes in urinary sediment, renal tissue oxygen saturation, Leiden’s surgical condition rating scale, surgery duration, and occurrence of bucking or body movement.

**Discussion:**

This study will provide evidence for the effect of LPP on renal function protection in patients with diabetes undergoing laparoscopic pelvic surgery. The trial can also help us to understand whether deep NMB can improve surgical conditions.

**Trial registration:**

ClinicalTrials.gov: NCT04259112. Prospectively registered on 5 February 2020.

## Background

Globally, the number of diabetes mellitus patients has quadrupled in the last 30 years, and China has the fastest growing epidemic of type 2 diabetes [1]. More than half of these patients suffer from at least one diabetic complication [2]. In China, the prevalence of diabetic kidney disease is as high as 24.3% in patients with type 2 diabetes [3], making it the leading cause of end-stage renal disease (ESRD) [4]. Renal failure accounts for approximately 10% of mortality in the presence of type 2 diabetes [5]; thus, the progression of diabetic nephropathy should be prevented as early as possible.

Postoperative acute kidney injury (AKI) affects 13–20% of patients undergoing major surgery [6, 7] and is associated with substantial morbidity and mortality [8, 9]. Pre-existing chronic kidney disease, including diabetic kidney disease, is associated with an increased risk of postoperative AKI [10]. Furthermore, diabetes is also an independent risk factor for postoperative AKI [11, 12]; thus, patients with diabetes deserve particular attention in terms of perioperative renal protection. According to the 2012 Kidney Disease: Improving Global Outcomes (KDIGO) criteria, AKI refers to the sudden decrease in renal function, defined by an elevation in serum creatinine (Cr) level or a decrease in urine output [13]. However, since an increase in Cr level may occur 2–3 days after renal ischemia [14], more sensitive biomarkers are necessary for the early recognition and prevention of AKI. Serum cystatin C (CysC) is one of these biomarkers [15]. In addition, renal oxygen saturation is also a promising predictor for AKI [16, 17].

Patients undergoing abdominal surgery are more likely to develop AKI than those undergoing other noncardiac, nonvascular surgeries [12, 18]. Pneumoperitoneum is necessary for better visualization and manipulation in laparoscopic surgery. The increased intra-abdominal pressure (IAP) induced by pneumoperitoneum can activate the renin-angiotensin-aldosterone system (RAAS) and cause renal vascular and parenchymal compression [19], which will further undermine renal perfusion and create ischemic insults [20]. Prior literature has demonstrated the association between increased IAP and AKI in critically ill patients after abdominal surgery [21, 22]. Intraoperative oliguria and diminished glomerular filtration rates have also been observed [23]. These changes deteriorate with increases in pressure and extended durations [23]. However, several studies have reported conflicting results, indicating that laparoscopic surgery does not carry an additional risk of postoperative AKI [24–26]. Therefore, high-quality evidence is needed for a better understanding.

Low-pressure pneumoperitoneum (LPP, 7–10 mmHg) is recommended to minimize the ischemic organ injury caused by standard-pressure pneumoperitoneum (SPP, 12–15 mmHg) [27]. LPP has been successfully used in a series of abdominal and pelvic surgeries, such as laparoscopic hysterectomy, cholecystectomy, and adrenalectomy [28–31]. However, LPP compromises the working space and surgical field, leading to prolonged surgical durations [32]. Fortunately, studies have shown that deep neuromuscular blockade (NMB) can ameliorate this issue [33, 34] and facilitate the use of LPP [35–37]. However, such effects are still controversial [38, 39]; thus, further studies are still required.

Therefore, the objective of this study is to assess the effect of LPP *versus* SPP on renal function protection in patients with diabetes undergoing laparoscopic pelvic surgery and to evaluate whether deep NMB can improve surgical conditions compared with moderate NMB. We hypothesize that the use of LPP, facilitated by deep NMB, can improve postoperative renal function after laparoscopic pelvic surgeries.

## Methods/design

### Study design

**Fig. 1:**
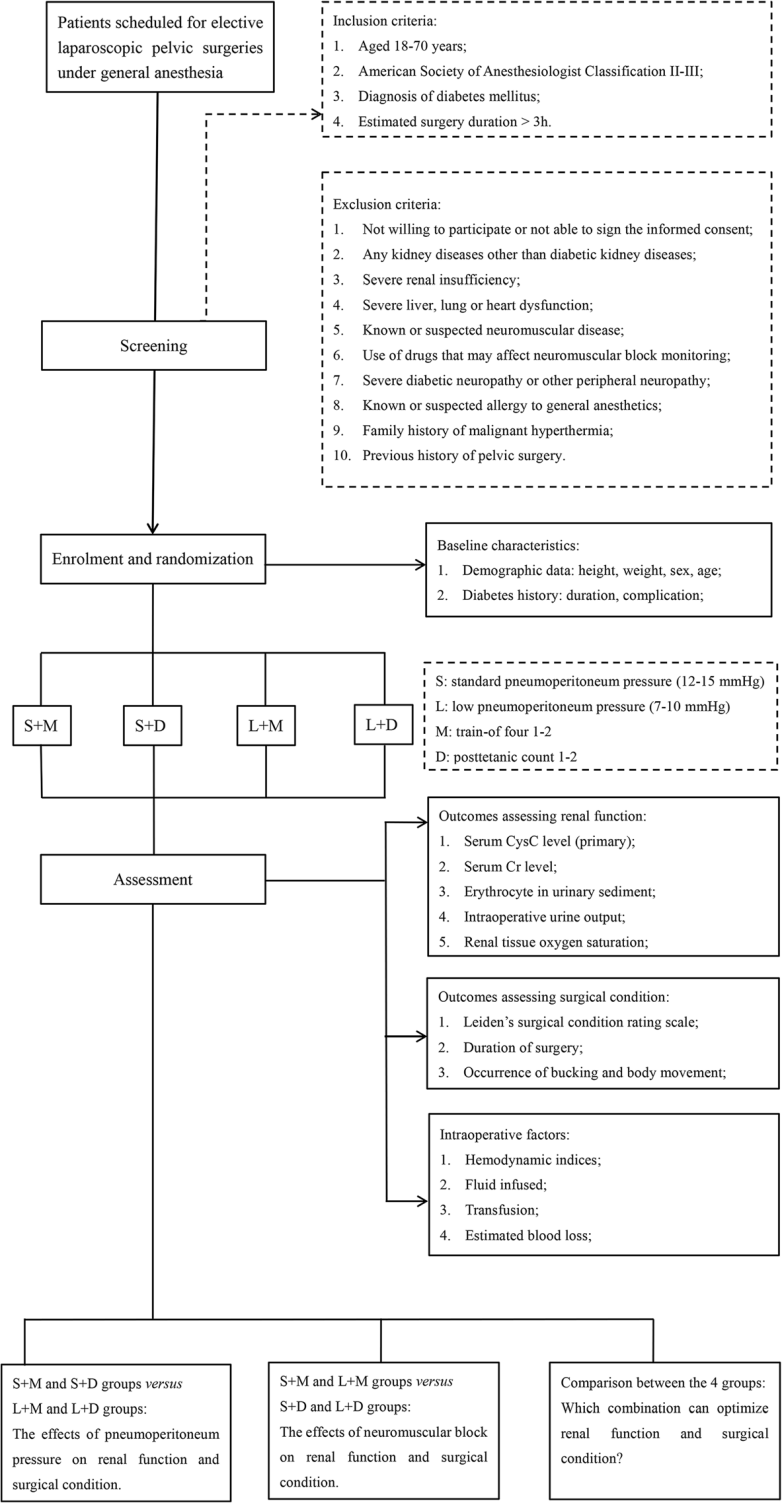
Flow chart of study design

This is a double-blinded, randomized controlled trial using a 2 × 2 factorial trial design, aiming to investigate the effect of pneumoperitoneum pressure and the depth of NMB on renal function in patients with diabetes undergoing laparoscopic pelvic surgery. Participants will be divided into the following four groups: (1) standard PP + deep NMB (S + D), (2) low PP + deep NMB (L + D), (3) standard PP + moderate NMB (S + M), and (4) low PP + moderate NMB (L + M) (Table 1). Deep NMB and moderate NMB are defined as a post-tetanic count (PTC) of 1–2 and a train-of-four (TOF) of 1–2, respectively. The pneumoperitoneum pressure will be set to 7–10 mmHg in the LPP groups and 12–15 mmHg in the SPP groups. We assume that no interaction is present between the two interventions being assessed on the primary outcome. The protocol is in accordance with the Standard Protocol Items: Recommendations for Interventional Trials (SPIRIT) guidelines (Additional file 1). The study flow chart is demonstrated in Fig. 1, and the overall schedule of enrollment, interventions, and assessments is shown in Table 1. All the members of the research team will be trained on the procedures prior to study commencement.

**Table 1:**
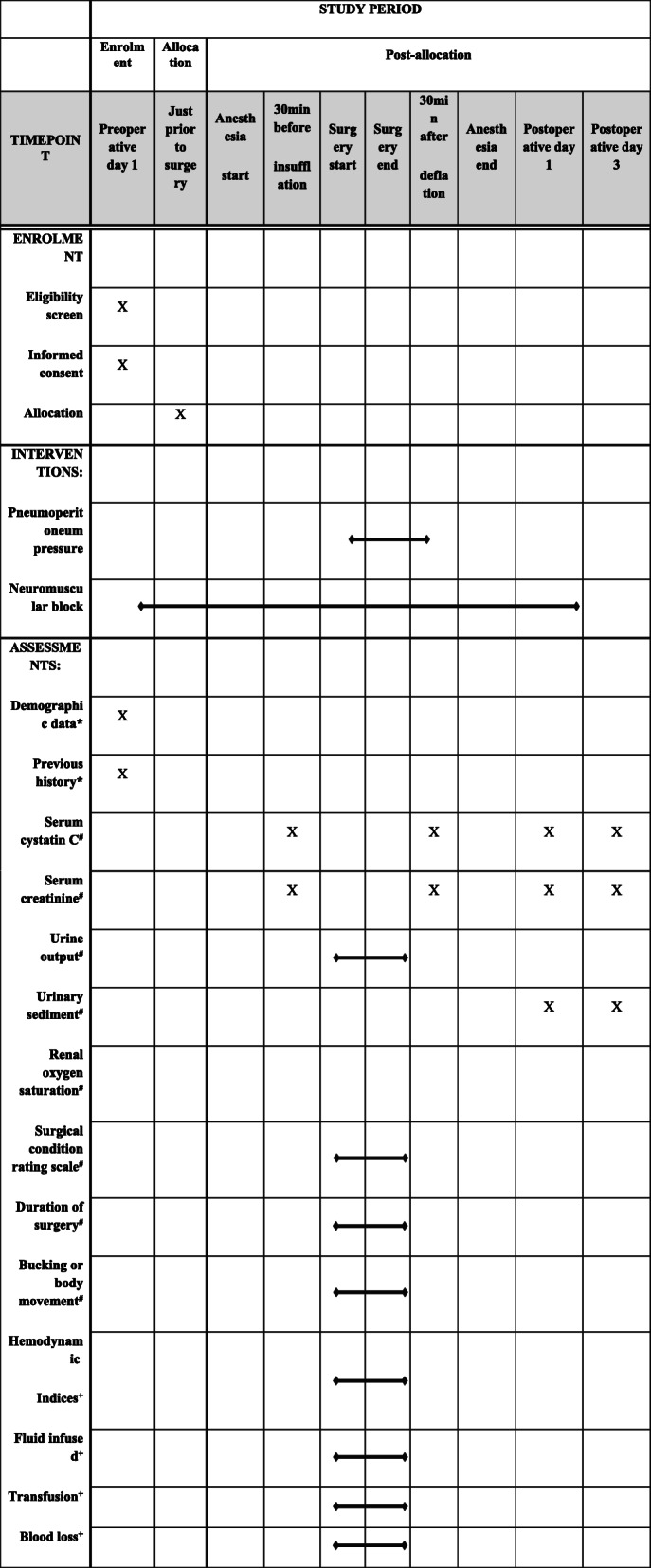
Schedule of enrollment, interventions, and assessments

^*^Baseline variables, ^#^outcome variables, ^**+**^intraoperative factors

### Settings

The study will be conducted in Peking Union Medical College Hospital (PUMCH), which is a tertiary general teaching hospital with 2200 beds in Beijing, China. An average of 20 elective pelvic surgeries are performed by five surgeons on each working day. The Ethics Committees of this hospital (reference number: ZS-1654) approved the trial.

### Participant selection

All patients scheduled for elective laparoscopic pelvic surgeries under general anesthesia will be screened for eligibility by an investigator who will not be involved in the process of randomization, intervention, data collection, or outcome assessment. Patients will be considered eligible if they meet the inclusion criteria and do not meet the exclusion criteria. Written informed consent will be obtained after evaluation.

The inclusion criteria are as follows:

- Age 18–70 years
- American Society of Anesthesiologist (ASA) Classification II-III
- Diagnosis of diabetes mellitus
- Estimated surgery duration > 3 h. The estimated surgery duration will be determined by the average duration of the same type of surgery by the same surgeon in the last year.

The exclusion criteria are as follows:

- Not willing to participate in the study or unable to sign informed consent
- Diagnosis of any kidney diseases other than diabetic kidney diseases
- Severe renal insufficiency defined as serum Cr > 2 times the upper limit of normal, urine output < 0.5 ml/kg/h, or estimated glomerular filtration rate (eGFR) < 60 ml/h
- Severe liver, lung or heart dysfunction
- Known or suspected neuromuscular disease
- Use of drugs that may affect neuromuscular block monitoring
- Severe diabetic neuropathy or any other peripheral neuropathy
- Known or suspected allergy to general anesthetics
- Family history of malignant hyperthermia
- Previous history of pelvic surgery

### Randomization and blinding

After enrollment, just prior to the surgery, the patients will be randomized by a statistician without the knowledge of random allocation in a balanced manner to one of the four groups. The allocation sequence will be computer-generated using random block sizes and will be kept in an opaque sealed envelope. These assignment envelopes will only be opened by a nurse, who will set the pneumoperitoneum pressure, prepare the muscle relaxant, and monitor the depth of NMB. Therefore, participants, anesthesiologists, surgeons, and outcome assessors will all be blinded to the allocation throughout the trial.

### Interventions

#### Anesthesia

An intravenous (IV) cannula will be inserted for the administration of anesthetic drugs. All enrolled patients will receive total intravenous anesthesia (TIVA). Intraoperative monitoring will include electrocardiography (ECG), pulse oximetry (SpO_2_), invasive arterial blood pressure (ABP), stroke volume (SV) and stroke volume variation (SVV) measured by LiDCO-rapid™, inspiratory and expiratory gas analysis, pressure of end tidal carbon dioxide (PetCO_2_), tidal volume, airway pressure, bispectral index (BIS), cerebral oxygen saturation, renal tissue oxygen saturation, nasopharyngeal temperature, and peripheral skin temperature. Anesthesia will be induced via the target-controlled infusion (TCI) of propofol (at an effect-site concentration of 6 μg/ml) and a bolus injection of 2–3 μg/kg fentanyl or 0.2–0.3 μg/kg sufentanil. Endotracheal intubation will be performed after the administration of a predefined dose of rocuronium based on the grouping. Anesthesia will be maintained with a TCI of propofol and continuous infusion of remifentanil. Mean blood pressure and heart rate fluctuations will be maintained within 20% of baseline, and the targeted BIS value will be 50–60. Fluid infusion will be adjusted to maintain SV < 10% and SVV < 13. Patients will be mechanically ventilated using a volume-controlled pattern with a tidal volume of 7–9 ml/kg and a frequency of 10–13/min. PetCO_2_ will be maintained between 35 and 45 mmHg. Forced-air warming will be used to maintain central and peripheral skin temperatures ≥ 36 °C and 32 °C, respectively.

#### Neuromuscular block monitoring and management

NMB will be monitored by acceleromyography at the adductor pollicis muscle using TOF-watch-SX™ (Organon, Oss, Netherlands) [40]. The arm without the IV cannula will be abducted and stabilized on an arm board. The thumb will be fixed to a flexible adaptor that will provide a constant preload. The other four fingers will be secured to minimize movement during nerve stimulation. After degreasing and cleansing of the skin with alcohol, two stimulating electrodes will be attached over the ulnar nerve, with the negative electrode on the wrist and the positive electrode placed 3–6 cm proximally. A transducer will be placed at the tip of the adductor pollicis. After the eyelash reflex disappears but before the administration of rocuronium, the TOF-watch-SX™ will be calibrated via the following steps. (1) A maximal response will be obtained using single-twitch stimulation (1 Hz for 0.2 ms) by gradually increasing the electrical current from 10 mA. (2) A supramaximal response will be triggered by an electrical current 15% above that necessary for a maximal response. (3) The TOF pattern (a set of four supramaximal stimuli at 2 Hz for 0.2 ms) will be applied to test the stability of the baseline response (variation in the TOF ratio < 5%) for 3 min. If the baseline response is not stable, the device will be recalibrated. Mask ventilation will be performed during calibration.

After successful calibration, 0.6 mg/kg rocuronium will be administered in the two moderate-NMB groups (S + M group and L + M group) and 1 mg/kg in the two deep-NMB groups (S + D group and L + D group). The TOF ratio will be measured every 1 min during anesthesia. When there is no response to TOF, the PTC pattern will be applied every 5 min, which is a single-twitch stimulation (2 Hz for 0.2 ms) starting 3 s after a tetanic stimulus (50 Hz for 5 s). In the two moderate-NMB groups, rocuronium will be continuously infused at an initial rate of 0.3 mg/kg/h, and the rate will be adjusted to maintain a TOF of 1–2. In the two deep-NMB groups, the infusion rate will be 0.6 mg/kg/h at first and then titrated for a targeted PTC of 1–2. Rocuronium infusion will be discontinued when pneumoperitoneum deflates.

Rocuronium (10 mg/ml) will be diluted by the nurse aware of the allocation. The concentration used at induction will be 0.1 × body weight (Wt) mg/ml in the deep-NMB groups and 0.06 × Wt mg/ml in the moderate-NMB groups; thus, 10 ml will be given in all four groups. The concentrations used for continuous infusion in the deep-NMB and moderate-NMB groups will be 0.06 × Wt mg/ml and 0.03 × Wt mg/ml, respectively; thus, the initial rate should be 10 ml/h. Therefore, one cannot tell the grouping from the injection dose or pump rate. An opaque curtain will be used to cover the NMB monitor. Only the nurse will be allowed to access the monitor and see the screen. If the NMB depth deviates from the target, the nurse will ask the anesthesiologist to adjust the pump rate.

#### Pneumoperitoneum pressure management

The nurse aware of the allocation will be the first to enter the operating room. She will initially set the pneumoperitoneum pressure at 7 mmHg in the LPP group and 12 mmHg in the SPP group. The pneumoperitoneum pressure can be increased to a maximum of 10 mmHg in the LPP group and 15 mmHg in the SPP group, if the surgery cannot proceed due to poor surgical sight. All the screens displaying intra-abdominal pressure will be covered with opaque tape; thus, the patients, surgeons, and data collectors will be blinded. The pneumoperitoneum pressure will remain unchanged during surgery.

#### Surgical condition rating

A well-validated, 5-point scale, Leiden’s surgical condition rating scale (Table 2) [33], will be used. The laparoscopic pelvic surgeries for the enrolled patients will be performed by five surgeons. Before the study begins, they will be asked to watch the videos demonstrating examples of scales 1–5.

**Table 2:** The surgical rating score [33]

Score	Impression	Description
1	Extremely poor conditions	The surgeon is unable to work because of coughing or because of the inability to obtain a visible laparoscopic field because of inadequate muscle relaxation. Additional neuromuscular blocking agents must be given.
2	Poor conditions	There is a visible laparoscopic field, but the surgeon is severely hampered by inadequate muscle relaxation with continuous muscle contractions, movements, or both with the hazard of tissue damage. Additional neuromuscular blocking agents must be given.
3	Acceptable conditions	There is a wide visible laparoscopic field but muscle contractions, movements, or both occur regularly causing some interference with the surgeon’s work. There is the need for additional neuromuscular blocking agents to prevent deterioration.
4	Good conditions	There is a wide laparoscopic working field with sporadic muscle contractions, movements, or both. There is no immediate need for additional neuromuscular blocking agents unless there is the fear of deterioration.
5	Optimal conditions	There is a wide visible laparoscopic working field without any movement or contractions. There is no need for additional neuromuscular blocking agents.

During the surgery, the surgeon will evaluate with the scale every 15 min starting from the introduction of the first trocar. At the same time, a 1-min video will be recorded by a camera connected to the endoscopic probe. The other four surgeons who will not perform this surgery will evaluate by watching videos independently. If the surgical condition suddenly worsens, additional assessments will be performed.

#### Postoperative management

Patients will stay in the postanesthesia care unit for 15–20 min and then return to the surgical ward, where they will spend at least 3 days before discharge. Nephrotoxic medications will be avoided in the postoperative period.

### Outcome measures

The primary outcome is serum CysC level, which is a sensitive biomarker of renal injury, and this will be measured 30 min before pneumoperitoneum insufflation (T1), 30 min after pneumoperitoneum deflation (T2), 24 h postoperatively (T3), and 72 h postoperatively (T4). Among them, T1 will be the baseline. The mean difference of the serum CysC between groups will be used as the effect measure. We will compare the serum CysC between groups throughout T2, T3, and T4, using mixed-effect model which can provide an estimate for the overall effect in the whole timeframe.

The secondary outcomes include four indicators of renal function: (1) serum Cr level measured at T1, T2, T3, and T4; (2) intraoperative urine output; (3) the presence of erythrocytes in urinary sediment on postoperative days 1 and 3; and (4) renal tissue oxygen saturation measured every 15 min following pneumoperitoneum insufflation, and cerebral oxygen saturation will be used as an internal reference to adjust individualized differences. The other three secondary outcomes selected to evaluate surgical conditions are (1) surgical condition rating scale measured every 15 min starting from the introduction of the first trocar by five surgeons independently, (2) duration of surgery, and (3) the occurrence of bucking or body movement during the surgery. The details of the secondary outcomes are demonstrated in Table 3.

**Table 3:** The secondary outcomes

Domain	Measurement	Metric	Aggregation	Time point
renal function	serum Cr level	difference between groups in the whole timeframe	mean	30 min before PP insufflation, 30 min after PP deflation, postoperative 24 h, and postoperative 72 h
renal function	intraoperative urine output	difference between groups	mean	NA
renal function	the presence of erythrocytes in urinary sediment	difference between groups	portion	postoperative day 1 and day 3
renal function	renal tissue oxygen saturation	difference between groups in the whole timeframe	mean	every 15 min since PP insufflation
surgical condition	surgical condition rating scale	difference between groups in the whole timeframe	median	every 15 min starting from the introduction of the first trocar
surgical condition	duration of surgery	difference between groups	mean	NA
surgical condition	the occurrence of bucking or body movement during surgery	difference between groups	portion	NA

*Abbreviations*: *NA* not applicable, *PP* pneumoperitoneum

### Data collection

The following demographic data and previous medical history will be collected by a data collector preoperatively from the medical records: sex, age, height, weight, duration of diabetes, and the presence of any complications of diabetes.

Peripheral venous blood samples will be extracted at T1, T2, T3, and T4, and urine samples will be obtained on the morning of postoperative days 1 and 3. The samples will be sent immediately to the clinical laboratory in PUMCH and will be analyzed by the staff there. Blood samples will be centrifuged at room temperature to obtain serum. Serum CysC concentration will be determined by sandwich enzyme-linked immunosorbent assay (ELISA), and Cr will be measured using an enzymatic method. Urine samples will be analyzed by automated urine microscopy analysis. All the samples will be handled within one hour since their arrival at the laboratory. We do not plan to store the samples after the study.

During the surgery, in addition to the secondary outcomes, two data collectors will record the heart rate, ABP, SV, and SVV every 15 min. They will also record the amount of crystalloid and colloid infused, the amount of transfusions, and the estimated blood loss.

There is an anesthesiologist and a surgeon in charge of monitoring the adherence to the protocol. Any suspected nonadherence during participant recruitment, randomization, intervention, data collection, or outcome assessment will be recorded. Based on the intention-to-treat (ITT) principle, all the randomized participants will be analyzed, despite any nonadherence to the protocol.

Data will be collected in the paper-based case report form (CRF) and then entered into a digital database by two independent investigators. Each patient will be distinguished by a unique serial number, and his or her name will be concealed. The database will be secured by a password, which will be restricted to the Data Safety Monitoring Committee (DMC), which is composed of two anesthesiologists with more than 10 years of experience. They will check the completeness and accuracy of the data, and the auditing will be independent from that of the other investigators. The information recorded in medical charts and saved in monitors will be traced to correct and supplement the data. The database will be locked up after the entry of the last case. The allocation will not be unblinded until all data analysis is completed; thus, the data analyst will also be blinded.

### Sample size calculation and statistical analysis

A previous study [15] reported that the standard deviation of serum CysC level after laparoscopic procedures is 0.738 ± 0.186 mg/dl. For a CysC difference of 0.05 mg/dl to be considered clinically significant, a power analysis program (G* power 3.1) calculated that a total sample size of 582 patients is needed to achieve 90% power at the 5% significance level. Considering a 10% dropout rate, a total of 648 patients (162 per group) is necessary. Approximately 5000 patients are scheduled for elective major pelvic surgery in PUMCH every year, and approximately 15% of them have diabetes; thus, one year is estimated as necessary to achieve adequate participant enrollment.

We will first compare the two SPP groups with the two LPP groups and then compare the two deep-NMB groups with the two moderate-NMB groups. Finally, all four groups will be compared.

The baseline characteristics and intraoperative factors of the enrolled patients will be evaluated using descriptive analysis. Continuous variables will be summarized as the mean  ±  standard deviation (SD) or median (interquartile range, IQR) as appropriate, whereas categorial variables will be summarized as the number (percentage). The baseline characteristics will be compared between groups using the standardized difference, and a standardized difference < 0.2 will be considered an acceptable balance between groups. Unbalanced variables at baseline will be included in the ANOVA or mixed-effects model to adjust for the potential confounding effect.

Considering the multiple measures of CysC, Cr, surgical condition rating scale, and renal tissue oxygen saturation at different time points, the effect of intervention in the whole timeframe will be analyzed by a mixed-effects model. Other continuous variables will be compared by two-sided independent Student’s t tests or the Mann–Whitney U test, as appropriate. Categorical variables will be analyzed by a Chi-square test or Fisher’s exact test, as appropriate. The Bonferroni method will be used to correct the potential increase in the probability of a type I error when analyzing the differences between the four groups.

We will also investigate the interaction between pneumoperitoneum pressure and NMB on postoperative renal function. The difference in serum Cysc levels at T2 between the S + D and L + D groups will be compared with that between the S + M and L + M groups. If there is no significant difference, we will assume there is no interaction.

We will perform statistical analysis using R (version 3.4.4). Two-sided *P* values < 0.05 will be considered significant.

Based on ITT principle, we will analyze all the post-randomization cases, including the ones with missing data. The missing data of the repeatedly measured outcomes will be imputed using the “last observation carried forward” method, while the missing data of other outcomes will be imputed by baseline data. Sensitivity analysis will be performed to assess the bias that may be introduced due to nonadherence to protocol or missing data.

### Adverse events

We anticipate that the potential risk of this study is minimal. First, to minimize the risks of failure in performing surgeries under LPP, we will exclude patients with a history of pelvic procedures, which may lead to adhesions and may increase the manipulation difficulty. Furthermore, all five surgeons involved have at least 10 years of experience in performing laparoscopic surgeries. Second, SPP has been safely used in PUMCH for more than 20 years. Patients with severe liver, heart, lung and kidney dysfunction will be excluded; thus, the potential risk of irreversible ischemic organ injury caused by SPP should be extremely low. Third, rocuronium is mainly metabolized in the liver, and no evidence exists to show that the dosage used in deep NMB has renal toxicity in patients without ESRD. Finally, to avoid residual paralysis, 6 mg neostigmine will be given at the end of surgery to antagonize the residual muscle relaxant, and patients will not be extubated until the TOF ratio is > 90%.

Adverse events information will be collected spontaneously from the participants during the follow-up and recorded in CRFs with details (time, severity, treatment, outcome, and the relationship to the intervention). Serious adverse events will be reported to the medical ethics committee.

## Discussion

To the best of our knowledge, this is the first trial to date assessing the effects of pneumoperitoneum pressure and NMB on postoperative renal function in diabetes patients. Accumulating evidence shows the benefits of combined LPP and deep NMB in reducing postoperative pain and enhancing recovery [28, 34]. However, several other studies have found negative results [41, 42]. Furthermore, previous research did not focus on renal function or diabetes patients. Considering the increasing prevalence of diabetes and the poor prognosis of renal complications, our study can provide important evidence on intraoperative renal protection in this high-risk population. Another strength of this study is that we plan to evaluate renal function using multiple biomarkers, including CysC and Cr levels, erythrocytes in urinary sediment, intraoperative urine output, and renal tissue oxygen saturation. We anticipate that their sensitivity will allow us to detect small changes in renal function. Furthermore, we will collect data on other intraoperative factors that may affect renal perfusion, such as hemodynamic indices and infused fluid, so we can test their balance and adjust potential confounding effects if necessary.

Our study is limited by the following aspects. First, we will exclude patients with ASA IV or with severe organ dysfunction; thus, the conclusion of this study cannot be generalized to critical cases. Second, since the surgeons involved in this study are experienced in laparoscopic procedures, they may have lower requirements for pneumoperitoneum pressure. Third, for the concern of workload, the diagnosis of diabetes will be based on the patients’ medical records rather than the “gold criteria,” the oral glucose tolerance test (OGTT). Therefore, some patients with undiagnosed early diabetes may be inadvertently excluded. Finally, unexpected events may occur, such as an actual surgery duration > 3 h or conversion to open surgery. Based on the ITT principle, the data of these cases should also be collected and analyzed. Considering the equal likelihood of the occurrence of non-adherence in each group, the effect on the results should be low.

In conclusion, our study will investigate the effects of LPP and deep NMB on postoperative renal function and surgical conditions. We hope this study can shed light on intraoperative renal protection in diabetes patients receiving laparoscopic surgeries.

## Trial status

The study protocol corresponds to the first version of the protocol, as submitted to *ClinicalTrials.gov* (URL: https://clinicaltrials.gov/ct2/show/NCT04259112, identifier: NCT04259112) on February 5, 2020. The recruitment will start on August 1, 2020, and will be completed on December 31, 2021. Data assessment should be completed in April 2022.

## Supplementary information

**Additional file 1.** Standard Protocol Items: Recommendations for Interventional Trials (SPIRIT) Checklist.

## Data Availability

All data generated or analyzed for the trial protocol are included in this published article. The data generated from the completed trial will be available from the corresponding author on reasonable request.

## Abbreviations

ESRD: End-stage renal disease
AKI: Acute kidney injury
KDIGO: Kidney Disease: Improving Global Outcomes
Cr: Creatinine
CysC: Cystatin C
IAP: Intra-abdominal pressure
RAAS: Renin-angiotensin-aldosterone system
LPP: Low-pressure pneumoperitoneum
SPP: Standard-pressure pneumoperitoneum
NMB: Neuromuscular blockade
S + D: Standard-pressure pneumoperitoneum + deep neuromuscular blockade group
L + D: Low-pressure pneumoperitoneum + deep neuromuscular blockade group
S + M: Standard-pressure pneumoperitoneum + moderate neuromuscular blockade group
L + M: Low-pressure pneumoperitoneum + moderate neuromuscular blockade group
PTC: Post-tetanic count
TOF: Train-of-four
SPIRIT: Standard Protocol Items: Recommendations for Interventional Trials
PUMCH: Peking Union Medical College Hospital
ASA: American Society of Anesthesiologist
eGFR: Estimated glomerular filtration rate
IV: Intravenous
TIVA: Total intravenous anesthesia
ECG: Electrocardiography
SpO_2_: Pulse oximetry
ABP: Arterial blood pressure
SV: Stroke volume
SVV: Stroke volume variation
PetCO_2_: Pressure of end tidal carbon dioxide
BIS: Bispectral index
TCI: Target-controlled infusion
Wt: Body weight
ELISA: Enzyme-linked immunosorbent assay
CRF: Case report form
ITT: Intention-to-treat
SD: Standard deviation
IQR: Interquartile range
OGTT: Oral glucose tolerance test
